# Physiological Chemistry

**Published:** 1891-01

**Authors:** John A. Miller


### PHYSIOLOGICAL CHEMISTRY.

CONDUCTED BY

JOHN A. MILLER, M. Sc., Ph. D.

### ABSORPTION OF FAT.

O. MINKOWSKI (*Practitioner*, 45, 139. *Jour. Chem. Soc.*, 58, 1171). From an examination of the feces, it was found that after extirpation of the pancreas the fat of the food is no longer absorbed. An exception to this was milk, whose fatty constituents were more or less taken up. By mixing the food with the fresh pancreas of the pig, the greater part of the fat was absorbed. The results lend no support to the view that the pancreatic juice breaks up fat into fatty acid and glycerine; on the contrary, the fatty matter in the feces was found, for the most part, split up, although the pancreas was entirely absent; this renders the theory that the fat is largely absorbed in the form of soaps untenable. The results show that the action of the juice is not a special one upon the absorbent elements of the intestines, as is evident from the fatty matter of milk being ingested in the absence of the gland, and they also show that what is *important* is the *form* in which the fat comes in contact with the intestinal mucous membrane.

The further observation was made that an action of the pancreas on fatty matters takes place, although there is no direct flow of the pancreatic secretion into the intestinal canal, provided a part of the pancreatic tissue capable of performing its functions remains in the organism. For example, after partial extirpation of the gland, fat absorption, though impaired, was not altogether prevented.

Another extremely interesting fact was also established, that in dogs complete removal of the pancreas leads to *diabetes;* and Hirschfeld has shown that in diabetes patients the absorption of proteid and fat is much impaired. This is, perhaps, an indication that the cause of diabetes in some cases is disease of the pancreas.

---

### DESTRUCTION OF GLUCOSE BY BLOOD AND CHYLE.

R. LEPINE and Barral (*Compt. rend.*, 110, 1314, *Jour. Chem. Soc.*; 58, 1172). Further experiments have been made by the authors, which confirm the existence in the blood, and especially in the chyle, of a ferment which destroys glucose. In cases of acute diabetes this ferment disappears. The activity of the ferment is increased by rise of temperature, but retarded by the presence of carbon dioxide. The experiments were made with dogs. The authors term the ferment the *glycolytic* ferment.

---

### PERNICIOUS ANEMIA.

F. W. MOTT (*Practitioner*, 45, 81. *Jour. Chem. Soc.* 58, 1177). The present case is remarkable as occurring in a young subject (*aet.* eleven years), in the fact that the urine, though loaded with uric acid crystals, was not highly colored, and in the presence of more iron in the spleen than has been previously noted. The amount of iron found in the various organs was as follows :

| Organ. | Wt. in grams. | Total iron as ferric oxide. |
|---|---|---|
| Liver | 900 | 1.33 grams. |
| Spleen | 100 | 0.17 " |
| Pancreas | 50 | 0.01 " |
| Kidneys | 100 | 0.01 " |

In connection with Delépine's work (next abstract) it is suggested that *pernicious anemia is an exaggeration of a normal physiological process.* When the absorption of the products of digestion occurs, the blood is replenished by new materials, and plethora would occur if there were not a proportional destruction of the plasma and corpuscles to form urea and pigments, which leave the body through natural channels. Whether the excessive destruction that occurs in pernicious anemia is due to a poison, the result of bacterial activity, derived from the alimentary canal, must, for the present, be considered unproved, for it does not seem that there is always a relation between the pyrexia, the anemia, and the color of the urine. Moreover, severe putrefactive processes may occur in the intestine without any pernicious anemia being brought about.

It is known that peptone has a very marked influence upon the blood, and it is possible to suppose that this substance, if not reconverted into albumin during absorption, might be the poison in question. The liver and spleen of a dog, into whose circulation peptone solution had been injected, yielded the iron reaction very markedly ; but this may have been accidental, and further experiments are promised on this subject.

---

### NORMAL STORAGE OF IRON IN THE LIVER.

S. Delépine (*Practitioner*, 45, 94. *Jour. Chem. Soc.*, 58, 1177). Pigment is constantly found in the liver cells and in some of the endothelial cells of the intralobular capillaries ; it may be particulate, or diffused through the cell in a state of solution. It gives the reactions of ferric salts, and these are more intense between eight and twelve hours after food has been taken, and reach a minimum immediately after the ingestion of food. The pigment is, as a rule, most abundant in the portal zone. The liver has thus a *ferrogenic* function, which probably consists of a separation of iron from effete iron-containing pigment, a storage of that iron in the form of a loose compound, and the gradual formation of a more stable albumin-ferruginous compound, analogous, if not identical, with hemoglobin, and ready for assimilation by the young red blood-corpuscles. Pernicious anemia is probably an exaggeration of this normal process.

